# Vagus nerve stimulation dampens intestinal inflammation in a murine model of experimental food allergy

**DOI:** 10.1111/all.13790

**Published:** 2019-04-15

**Authors:** Goele Bosmans, Iris Appeltans, Nathalie Stakenborg, Pedro J. Gomez‐Pinilla, Morgane V. Florens, Javier Aguilera‐Lizarraga, Gianluca Matteoli, Guy E. Boeckxstaens

**Affiliations:** ^1^ Translational Research Center for Gastrointestinal Disorders (TARGID) Department of Chronic Diseases, Metabolism and Ageing (CHROMETA) KU Leuven Leuven Belgium

**Keywords:** cholinergic anti‐inflammatory pathway, CX3CR1 macrophages, food allergy, mast cells, vagus nerve stimulation

## Abstract

**Background:**

The vagus nerve has emerged as an important modulator of the intestinal immune system. Its anti‐inflammatory properties have been previously shown in innate and Th1/Th17 predominant inflammatory models. To what extent the vagus nerve is of importance in Th2 inflammatory responses like food allergy is still unclear. In this study, we therefore aimed to investigate the effect of vagotomy (VGX) and vagus nerve stimulation (VNS), on the development and severity of experimental food allergy.

**Methods:**

Balb/C mice were first sensitized with ovalbumin (OVA) in the presence of alum. Prior to oral challenges with OVA, mice were subjected to VGX or VNS. Disease severity was determined by assessing severity and onset of diarrhoea, OVA‐specific antibody production, mast cell number and activity, inflammatory gene expression in duodenal tissue and lamina propria immune cells by flow cytometry analysis.

**Results:**

When compared to control mice, VGX did not significantly affect the development and severity of the disease in our model of food allergy. VNS, on the other hand, resulted in a significant amelioration of the different inflammatory parameters assessed. This effect was independent of α7nAChR and is possibly mediated through the dampening of mast cells and increased phagocytosis of OVA by CX3CR1^hi^ macrophages.

**Conclusions:**

These results underscore the anti‐inflammatory properties of the vagus nerve and the potential of neuro‐immune interactions in the intestine. Further insight into the underlying mechanisms could ultimately lead to novel therapeutic approaches in the treatment of not only food allergy but also other immune‐mediated diseases.

## Graphical abstract

Vagus nerve stimulation (VNS) has anti‐inflammatory effects in a Th2 inflammatory model of experimental food allergy. VNS dampens the intestinal inflammatory response as evidenced by reduced expression of inflammatory genes IL‐4, IL‐5, IL‐13 and IL‐6, reduced neutrophil infiltration and reduced number and activation of mast cells. Phagocytosis of allergen by macrophages is increased in response to VNS. The contrary is observed for eosinophils. ACh: acetylcholine

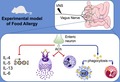

## 1 INTRODUCTION

Food allergy has been on the rise in westernized countries for several decades now, with numbers reportedly rising up to approximately 10%. To date, no effective treatment exists, and management of this disorder is largely based on avoidance of allergenic foods.^1^ Food allergy patients fail to develop oral tolerance towards innocuous food antigens, which results into the establishment of an aberrant type 2 immune response. The release of epithelial cytokines, IL‐25, IL‐33 and thymic stromal lymphopoietin (TSLP) in response to allergen exposure is thought to play an important role in the initiation of allergic responses, as these cytokines are able to induce expression and release of type 2 cytokines by type 2 innate lymphoid cells (ILC2s).^2^ This creates an environment that promotes the generation of allergen‐specific Th2 cells, which in turn can orchestrate different phases of the inflammatory allergic response, mainly through the release of type 2 cytokines such as IL‐4, IL‐5, IL‐9 and IL‐13. These phases include the attraction of effector cells including eosinophils and mast cells (MCs), and production of allergen‐specific immunoglobulin E (IgE) by B cells. Allergen re‐exposure results in IgE‐mediated MC activation, with MCs releasing a range of harmful mediators leading to the typical symptoms observed in food allergy.^3^, ^4^

The importance of the bidirectional relationship between the nervous system and the immune system in both homeostasis and inflammatory conditions has become increasingly clear in recent years. In 2000, Tracey et al^5^ introduced the “inflammatory reflex,” showing the ability of the vagus nerve (VN) to sense peripheral inflammation and inform the brain on the inflammatory status to then subsequently dampen the inflammatory response. This concept was later translated to the gastrointestinal tract, showing that vagal nerve stimulation (VNS) reduced inflammation of the intestinal muscle layer and restored gastrointestinal transit in a model of postoperative ileus.^6^ Furthermore, it was demonstrated this effect is exerted by activation of enteric cholinergic neurons interacting with resident muscular CX3CR1^hi^ macrophages via α7 nicotinic acetylcholine receptors (α7nAChR).^7^ To date, several additional studies have evaluated the effect of VNS and pharmacological cholinergic modulation in a range of experimental inflammatory models including endotoxemia, sepsis, inflammatory bowel disease, obesity and arthritis.^8^, ^9^, ^10^, ^11^, ^12^, ^13^, ^14^

We recently showed vagotomized mice fail to develop oral tolerance, indicating an important modulatory role for the VN in inducing and preserving oral tolerance induction and therefore the maintenance of intestinal homeostasis.^15^ Considering that loss of intestinal immunological tolerance towards food antigens is typically associated with food allergy, we hypothesize that cholinergic input could have a significant impact on the induction and course of this disease. In this study, we therefore aimed to investigate the effect of both reduced and increased cholinergic input, by means of vagotomy (VGX) and VNS, on the development and severity of experimental food allergy. Elucidating a potential role for cholinergic modulation in type 2‐mediated food allergy could provide novel insights to increase our understanding in the pathogenesis of food allergy and ultimately lead to development of new treatment options for food allergy.

## 2 METHODS

### 2.1 Mice

All mice were bred and maintained under specific pathogen‐free conditions at the KU Leuven animal facility until the start of each experiment. α7nAChR^−/−^ (B6.129S7‐*Chrna* ^*7tm1Bay*^/J) and IL‐4^gfp^ (4get, C.129‐*Il4* ^*tm1Lky*^/J) were purchased from The Jackson Laboratory, Bar Harbor, ME. B6.129S7‐*Chrna* ^*7tm1Bay*^/J were backcrossed into a Balb/c background (F8), to obtain α7nAChR^−/−^ Balb/c mice. CX3CR1^gfp/wt^ Balb/c (C.129P2‐*Cx3cr1* ^*tm1Litt*^/Ieg) were kindly provided by Stephen Jung via the European Mouse Mutant Archive (EMMA). Mice were housed with ad libitum access to standard rodent food and water and genotyped using PCR of DNA isolated from ear punch biopsies. All experimental procedures were approved by the Animal Care and Animal Experiments Committee of the KU Leuven, Leuven, Belgium.

### 2.2 Experimental food allergy

Mice (6‐8 weeks) were sensitized two times (day 0 and 14) with 100 μg of OVA in the presence of 1 mg alum by intraperitoneal injection. From day 28 onwards, animals were challenged every other day by oral administration of 50 mg OVA in 200 μL PBS. Before each intragastric challenge, mice were fasted for 3‐4 hours to limit antigen degradation in the stomach. Mice were monitored for up to 1 hour following intragastric challenge with OVA. Timing of onset and severity of diarrhoea was assessed.

### 2.3 Vagotomy

Mice were anaesthetized by intraperitoneal (i.p.) injection of a mixture of Ketamine (Ketalar 100 mg/kg; Pfizer) and Xylazine (Rompun 10 mg/kg; Bayer). The skin and abdominal wall were incised along the ventral midline, and the intestines were moved aside to allow access to the left lateral lobe of the liver and the stomach. The left lateral lobe of the liver was gently retracted, and the stomach pulled down beneath the diaphragm to clearly expose both vagal trunks, which were then transected. All neural and connective tissue surrounding the oesophagus was removed to ensure transection of all small vagal branches. Pyloroplasty (PP) was used to avoid gastric dilatation due to VGX. As control mice, we performed sham operation, PP was performed and vagal trunks were exposed but not cut.

### 2.4 Vagal nerve stimulation

Anaesthetized (2.5% isoflurane) (ISO‐VET, Eurovet NV/SA) mice underwent surgery and VNS or sham stimulation. A ventral midline cervical incision was made between the mandible and sternum; subcutaneous tissue was dissected and retracted laterally. The mandibular salivary glands were bluntly separated and retracted laterally. The right cervical VN was isolated from the carotid artery and stimulated electrically using a bipolar platinum electrode (Bilaney). Electrical stimuli consisted of square pulses with a frequency of 10 Hz, current of 1 mA and a pulse width of 1 ms (Keithley Instruments) for 5 minutes. Sham‐operated mice were handled similarly, but the VN was not dissected from the carotid artery to avoid mechanical stimulation.

### 2.5 Flow cytometry analysis

Lamina propria single cell suspensions obtained from small intestines were first blocked with anti‐CD16/CD32 (2.4G2, BD Biosciences) before staining with various fluorescently labelled antibodies (Table S1). Biotinylated antibodies were further treated with FITC or PeCy7‐labelled streptavidin (eBioscience). Dead cells were excluded by staining with Fixable Viability Dye eFluor450 (eBioscience). Data were acquired on a 3 laser, 8 colour custom‐configuration FACS Canto II flow cytometer (BD Biosciences) and analysed by FlowJo software (Tree Star).

### 2.6 Real‐time quantitative PCR

Total RNA was isolated from duodenal tissue using RNeasy Mini Kit (Qiagen) following the manufacturer's instructions. Total RNA was transcribed into complementary DNA (cDNA) by qScript cDNA SuperMix (Quanta Biosciences) according to the manufacturer's instructions. Quantitative real‐time transcription polymerase chain reactions were performed with the LightCycler 480 SYBR Green I Master on the Light Cycler 480 (Roche). Results were quantified using the 2^−ΔΔCT^ method. Expression levels of the genes of interest were normalized to the expression levels of the reference gene Rpl32. Data are expressed as fold induction vs naive controls. Primer sequences used are listed in Table S2.

### 2.7 ELISA measurements

mMCP‐1 and OVA‐specific IgE levels were measured by ELISA following manufacturer's instructions (eBioscience and BioLegend, respectively). For OVA‐specific IgG1 and IgG2a levels, an in‐house ELISA was developed. In brief, micro‐titre plates (NUNC Maxisorp, Thermo Scientific) were coated with 5 μg/mL OVA grade V (Sigma‐Aldrich) in carbonate buffer and incubated overnight and then blocked for 1 hour with 3% BSA in PBS, before adding serial dilutions of plasma samples on the plates. After 1 hour incubation, detection antibody, 5 μg/mL biotin‐conjugated rat anti‐mouse IgG1 (LO‐MG1‐2, Bio‐Rad) or 1 μg/mL biotin‐conjugated rat anti‐mouse IgG2a (R19‐15, BD biosciences) was added and incubated for 1 hour. Thereafter, streptavidin‐HRP (R&D systems), diluted 1:200, was added. Before the initiation of each step, plates were washed 5 times with 0.05% of Tween‐20 in PBS. Finally, after 1 hour of incubation, TMB substrate solution (eBioscience) was added. The colorimetric reaction was stopped with 2M H_2_SO_4_, and the absorbance was measured at a wavelength of 450 nm with an ELISA plate reader.

### 2.8 Ussing chambers

Freshly isolated duodenal tissue was mounted in Ussing chambers (Mussler Scientific Instruments) with a 0.017 cm^2^ opening and without removal of the seromuscular layer. Transepithelial electrical resistance (open circuit conditions, bipolar constant‐current pulses 16 μA, 200 ms) and paracellular passage of FITC‐dextran (4 kDa, Sigma‐Aldrich) were measured every 30 minutes over a period of 180 minutes. The degree of fluorescence was measured using a FLUOstar Omega fluorescence reader (BMG Labtech) and the transepithelial resistance with Clamp software version 9.00 (Mussler Scientific Instruments).

### 2.9 Statistics

Normality was determined via the Shapiro‐Wilk test prior to statistical analysis. Two‐way analysis of variance (ANOVA) followed by Bonferroni post hoc test was performed to compare multiple groups and multiple variables. To compare two independent groups and a single variable, the unpaired *t* test was performed. To compare categorical variables, the *χ* ^2^ test was used. Probability level of *P *<* *0.05 was considered statistically significant. GraphPad Prism software was used to perform statistical analysis.

## 3 RESULTS

### 3.1 Vagotomy did not significantly affect the severity of experimental food allergy

**Figure 1:**
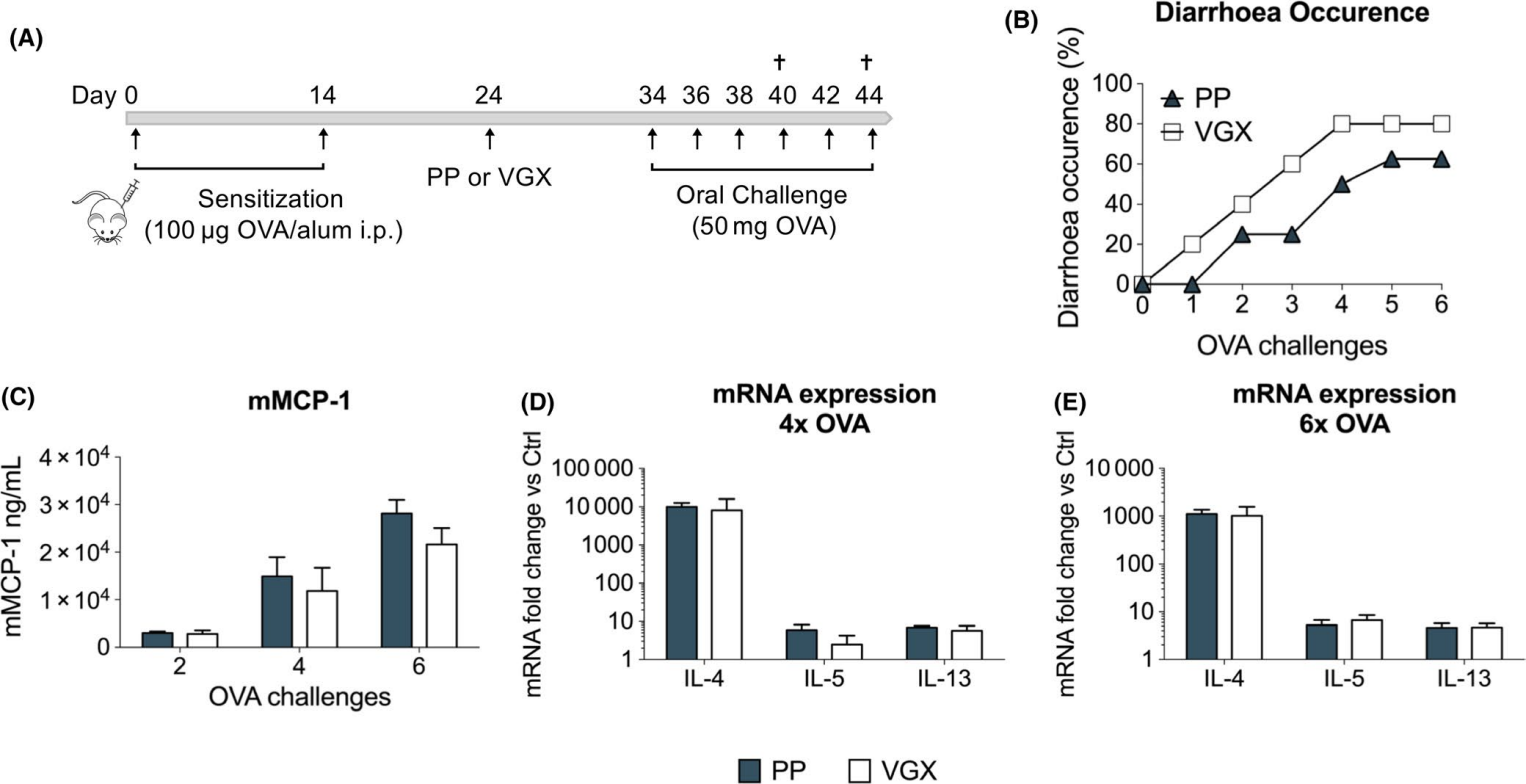
Vagotomy did not significantly affect the severity of experimental food allergy. A, Balb/C mice were sensitized with two OVA/alum intraperitoneal injections (day 0 and 14). On day 24, mice were subjected to pyloroplasty (PP) or vagotomy (VGX), and subsequently received oral OVA challenges every other day from day 34 onwards. B, Diarrhoea occurrence was monitored in PP‐ and VGX‐treated mice following six oral OVA challenges. C, mMCP‐1 plasma levels were measured by ELISA following two, four and six OVA challenges. D‐E, mRNA expression of Th2‐associated genes quantified in duodenal tissue in PP‐ and VGX‐treated mice following (D) four and (E) six OVA challenges. Error bars denote mean ± SEM (n = 5‐8). Statistical significance was determined with *χ* ^2^ test in (B), 2‐way ANOVA in (C) and unpaired *t* test in (D) and (E) [Color figure can be viewed at http://wileyonlinelibrary.com]

In mice sensitized to OVA, re‐exposure to OVA initiates an inflammatory process in the gut, which is further potentiated with every challenge, typically resulting in intestinal symptoms like diarrhoea. To study the role of vagal tone in the development of food allergy, we first assessed the impact of VGX on the development of allergic diarrhoea (Figure 1A,B). Though VGX mice developed more often diarrhoea (80%) compared to PP control mice (63%) (Figure 1B), this difference was not significant. As the development of diarrhoea is MC mediated, we investigated whether VGX had an impact on serum levels of mMCP‐1, a marker of MC degranulation. As shown in Figure 1C, the increase in mMCP‐1 levels induced by repetitive OVA challenges did not significantly differ in VGX mice compared to PP mice at any of the timepoints measured. Additionally, duodenal expression of Th2 genes was not significantly altered by VGX (Figure 1D‐E). These findings show that an impaired vagal tone does not significantly affect the severity of experimental food allergy, suggesting that the basal vagal tone is redundant under these conditions.

### 3.2 Vagus nerve stimulation improves experimental food allergy

**Figure 2:**
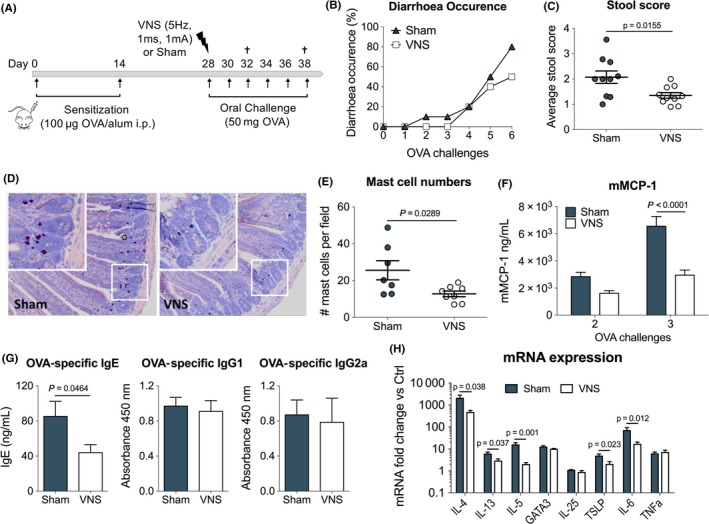
Vagus nerve stimulation improves experimental food allergy. A, Balb/C mice were sensitized with two OVA/alum intraperitoneal injections (days 0 and 14). On day 28, mice received sham or VNS treatment prior to oral OVA challenge. From that day onward, mice were further challenged with OVA every other day. B, Diarrhoea occurrence and C, stool score were monitored in sham‐ and VNS‐stimulated mice following six oral OVA challenges. D, Representative duodenum sections from sham‐ and VNS‐stimulated mice stained with CAE staining and (E) intestinal mast cell numbers following three oral challenges. F, mMCP‐1 plasma levels were measured by ELISA following two and three OVA challenges. G, OVA‐specific IgE, IgG1 and IgG2a plasma titres were measured following three OVA challenges. H, mRNA expression of Th2‐associated and inflammatory genes quantified in duodenal tissue in sham‐ and VNS‐stimulated mice following three OVA challenges. Error bars denote mean ± SEM (n = 7‐8). Statistical significance was determined with unpaired *t* test in (C, E, G, H) and with 2‐way ANOVA in (F)

As we previously showed anti‐inflammatory properties of VNS in colitis and postoperative ileus, we next evaluated the role of VNS on the development of food allergy.^14^, ^16^ To this end, OVA‐sensitized Balb/C mice received VNS or sham stimulations prior to the first oral OVA challenge (Figure 2A). Mice that underwent VNS exhibited a lower incidence of allergic diarrhoea (50%) compared to sham mice (80%) and showed significant improvement in stool scores following six OVA challenges (Figure 2B‐C). The number of MCs present in the duodenum was evaluated by chloracetate esterase (CAE) staining and was significantly decreased in VNS‐treated mice (Figure 2D‐E). This coincided with significantly lower serum levels of mMCP‐1 (Figure 2F). In addition, serum OVA‐specific IgE antibodies, but not OVA‐specific IgG1 and IgG2a antibodies, were significantly reduced by VNS. In line, expression levels of Th2‐associated and inflammatory genes in duodenal tissue, including IL‐4, IL‐5, IL‐13 and TSLP and IL‐6, were significantly reduced by VNS (Figure 2H). Together, these data show the ability of VNS to significantly dampen the inflammatory response in an experimental model Th2‐mediated food allergy.

### 3.3 Vagus nerve stimulation reduces neutrophil infiltration and mastocytosis

**Figure 3:**
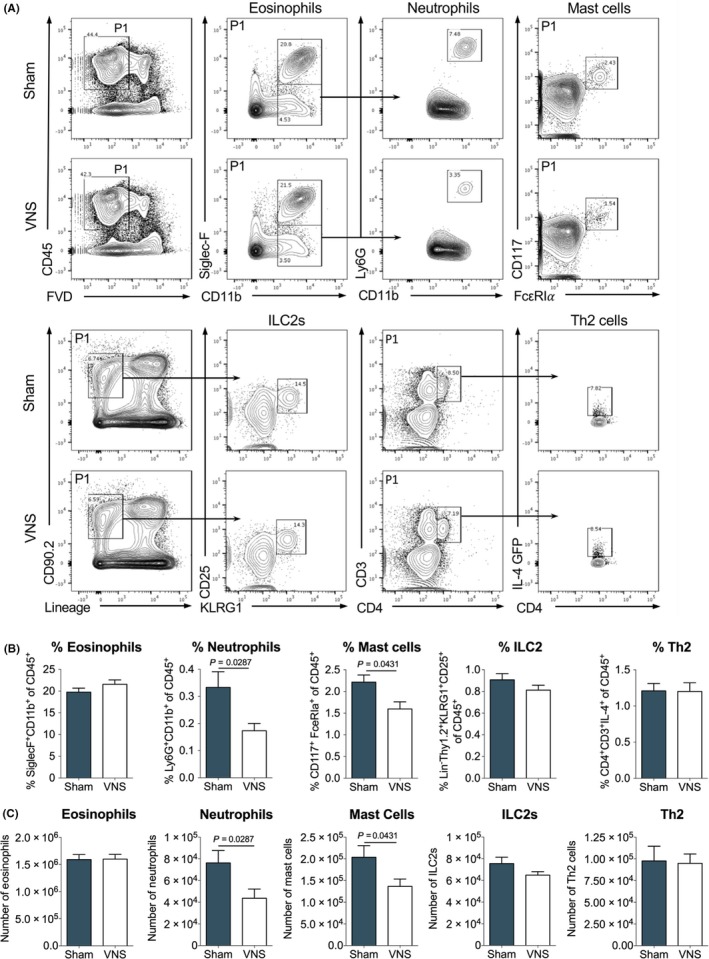
Vagus nerve stimulation reduces neutrophil infiltration and mastocytosis in experimental food allergy. A, Flow cytometry analysis of CD45^+^ SI LP eosinophils (CD11b^+^ Siglec‐F^+^), neutrophils (CD11b^+^ Siglec‐F^−^ Ly6G^+^), mast cells (CD117^+^ Fc**ε** RIα^+^), ILC2s (Lin^−^ CD90.2^+^ KLRG1^+^ CD25^+^) and Th2 cells (CD3^+^ CD4^+^ IL4^+^) in sham‐ and VNS‐treated mice following three OVA challenges. B‐C, Data are shown as (B) frequency of CD45^+^ and (C) absolute numbers. Error bars denote mean ± SEM (n = 7‐12). Statistical significance was determined with unpaired *t* test

Given the pronounced beneficial effect of VNS on the different inflammatory parameters assessed, we next investigated the cell populations affected by VNS. Therefore, we performed an extensive flow cytometry analysis on several immune cell populations within the lamina propria (LP) of the small intestine (SI) (Figure 3A). To this end, we took advantage of the bicistronic *Il4* ^*gfp*^ reporter mice (4get), expressing eGFP in IL‐4 expressing cells, a key cytokine in allergic responses. OVA‐sensitized *Il4* ^*gfp/+*^ mice were sham‐ or VNS‐stimulated as before and immune cells were isolated from the LP following three oral OVA challenges. Comparison of the percentages and absolute number of eosinophils, neutrophils, MCs, ILC2s and Th2 cells in the SI LP of VNS‐ and sham‐stimulated mice revealed no significant differences in eosinophils, ILC2s and Th2 cells. However, the percentage and absolute number of MCs in the LP of VNS‐stimulated mice was significantly reduced, in line with earlier results obtained by CAE staining on SI tissue. Additionally, VNS inhibited the infiltration of neutrophils as indicated by a significantly reduced percentage and number of neutrophils in the LP. These results further support the ability of VNS to efficiently reduce inflammation in food allergy.

### 3.4 Vagus nerve stimulation improves experimental food allergy independently of α7nAChR

**Figure 4:**
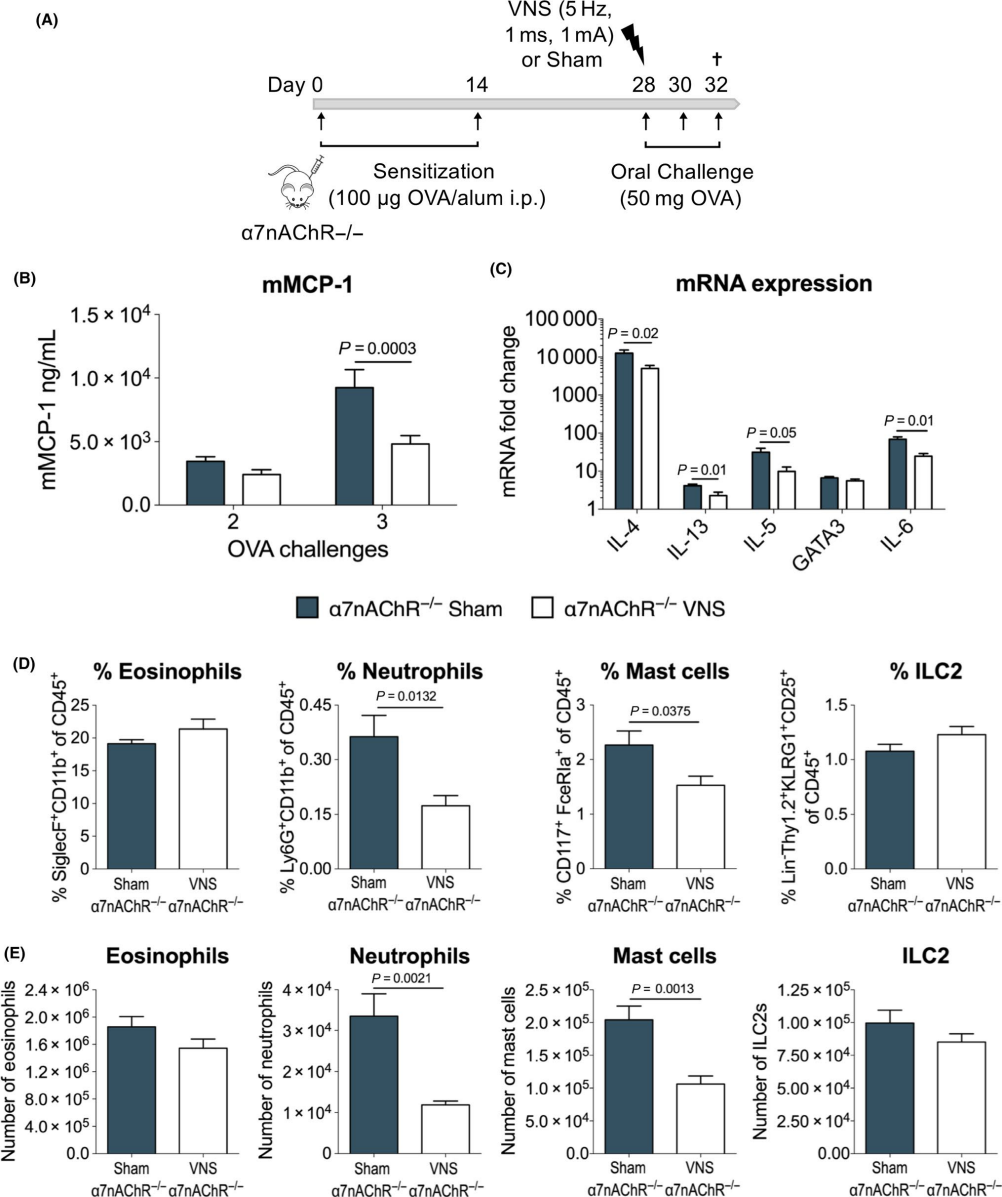
Vagus nerve stimulation improves experimental food allergy independently of α7nAChR. A, α7nAChR^−/−^ mice were sensitized with two OVA/alum intraperitoneal injections (days 0 and 14). On day 28, mice received sham or VNS treatment prior to oral OVA challenge. From that day onward, mice were further challenged with OVA every other day. B, mMCP‐1 plasma levels were measured by ELISA following two and three OVA challenges. C, mRNA expression of Th2‐associated and inflammatory genes quantified in duodenal tissue in sham‐ and VNS‐stimulated α7nAChR^−/−^ mice following three OVA challenges. D‐E, Flow cytometry analysis of CD45^+^ SI LP eosinophils (CD11b^+^ Siglec‐F^+^), neutrophils (CD11b^+^ Siglec‐F^−^ Ly6G^+^), mast cells (CD117^+^ Fc**ε** RIα^+^), ILC2s (Lin^−^ CD90.2^+^ KLRG1^+^ CD25^+^) in sham‐ and VNS‐treated α7nAChR^−/−^ mice following three OVA challenges. Data are shown as (D) frequency of CD45^+^ and (E) absolute numbers. Error bars denote mean ± SEM (n = 8‐9). Statistical significance was determined with 2‐way ANOVA in (B) and unpaired *t* test in (C, D, E) [Color figure can be viewed at http://wileyonlinelibrary.com]

The critical involvement of the nicotinic acetylcholine receptor α7nAChR has been shown in the anti‐inflammatory effect of the VN in sepsis, a model of systemic inflammation, and in a model of intestinal inflammation, that is postoperative ileus.^7^ To investigate the role of α7nAChR in the anti‐inflammatory effect of VNS in food allergy, VNS or sham stimulation was performed in OVA‐sensitized α7nAChR^−/−^ mice as earlier described, prior to the first challenge with OVA (Figure 4A). Of note, similar to wild‐type mice (Figures 2 and 3), VNS significantly decreased mMCP‐1 (Figure 4B) and Th2‐associated and inflammatory genes (IL‐4, IL‐5, IL‐13 and IL‐6) (Figure 4C) compared to sham stimulation in α7nAChR^−/−^ mice. Furthermore, VNS reduced the percentage and total number of neutrophils and MCs in the SI LP in α7nAChR^−/−^ mice compared to sham stimulation (Figure 4D). These data further confirm the anti‐inflammatory effect of VNS in food allergy; however, this effect is independent of the α7nAChR.

### 3.5 Vagus nerve stimulation does not significantly alter small intestinal permeability

**Figure 5:**
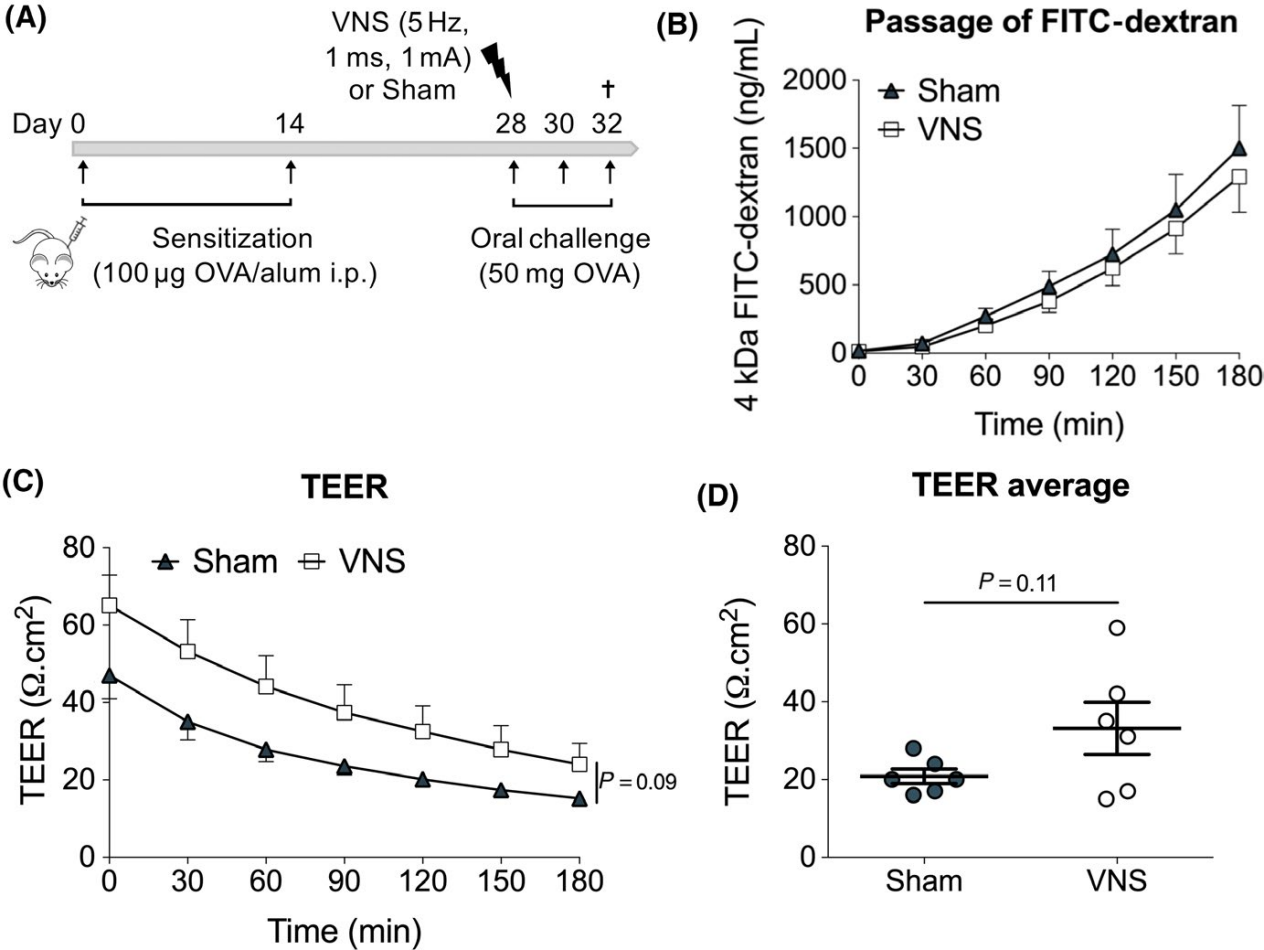
Vagus nerve stimulation does not significantly alter SI permeability. A, Balb/C mice were sensitized with two OVA/alum intraperitoneal injections (day 0 and 14). On day 28, mice received sham or VNS treatment prior to oral OVA challenge. From that day onward, mice were further challenged with OVA every other day. Intestinal permeability was assessed by Ussing chamber experiments 1 h after the third OVA challenge. B, Passage of FITC‐dextran and (C‐D) TEER in sham‐ and VNS‐treated mice after three oral OVA challenges. Error bars denote mean ± SEM (n = 6) Statistical significance was determined with 2‐way ANOVA in (B, C) and unpaired *t* test in (D)

Activation of MCs results from crosslinking of IgE antibodies by binding to antigens. As the access of antigens is strongly dependent on barrier integrity, and cholinergic modulation has been proposed to increase barrier integrity, we next evaluated the effect of VNS on SI permeability using Ussing chambers. As shown in Figure 5A, VNS did not alter the passage of 4kDA FITC‐dextran. Furthermore, although transepithelial resistance (TEER) was increased by VNS mice, this difference did not reach statistical significance (Figure 5C‐D). Together, these data suggest no significant improvement of SI barrier function in response to VNS.

### 3.6 Vagal nerve stimulation alters OVA distribution in immune cells

**Figure 6:**
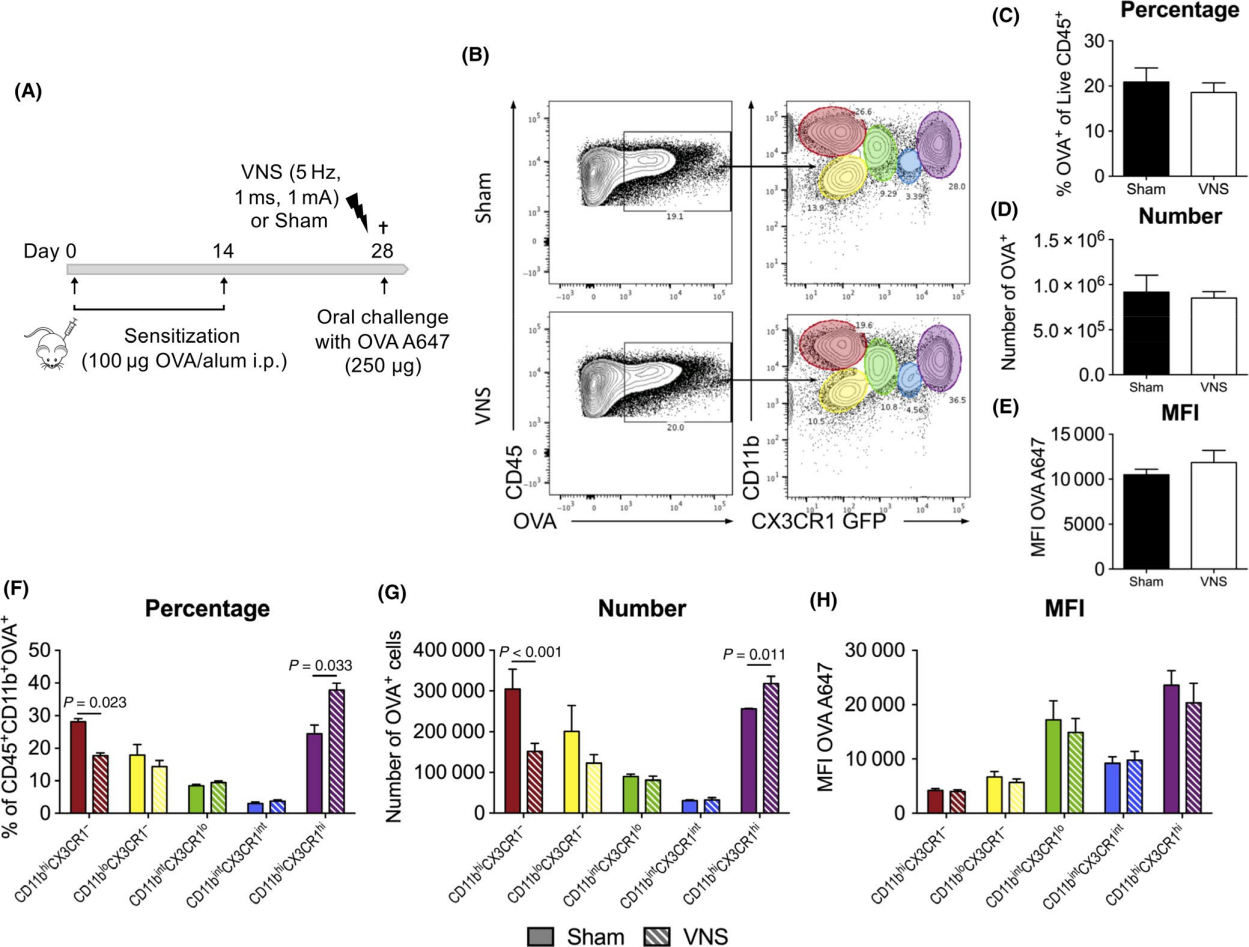
Vagus nerve stimulation alters OVA distribution in immune cells. A, Balb/C mice were sensitized with two OVA/alum intraperitoneal injections (days 0 and 14). On day 28, mice received sham or VNS treatment prior to oral challenge with fluorescently labelled Alexa Fluor^®^ 647 OVA. Duodenal tissue was collected, and SI LP cells were isolated 15 min after the challenge. B, Flow cytometry analysis of CD45^+^ OVA ^+^ SI LP subpopulations in sham‐ and VNS‐treated mice. CD11b and CX3CR1 expression was used to identify five different populations, CD11b^hi^ CX3CR1^−^ (red), CD11b^lo^ CX3CR1^−^ (yellow), CD11b^int^ CX3CR1^lo^ (green), CD11b^int^ CX3CR1^int^ (blue) and CD11b^hi^ CX3CR1^hi^ (purple). C‐E, Data for OVA ^+^ SI LP immune cells are represented as (C) frequency of CD45^+^, (D) absolute numbers and (E) mean fluorescence intensity levels of Alexa Fluor^®^ 647. F‐H, Data for OVA ^+^ SI LP subpopulations are represented as (F) frequency of pregate (CD45^+^ OVA ^+^), (G) absolute numbers and (H) mean fluorescence intensity levels of Alexa Fluor^®^ 647. Error bars denote mean ± SEM (n = 3‐4). Statistical significance was determined with unpaired *t* test [Color figure can be viewed at http://wileyonlinelibrary.com]

As VNS did not significantly affect barrier function, we hypothesized that increased clearance or uptake of OVA by immune cells in the LP could contribute to the beneficial effect of VNS. To this end, fluorescently labelled Alexa Fluor^®^ 647 ovalbumin was administered to OVA‐sensitized mice treated with VNS or sham stimulation (Figure 6A). Immune cells were isolated from the SI LP 15 minutes after challenge, and the distribution of OVA A647 in immune cells was analysed by flow cytometry (Figure 6B). Both VNS‐ and sham‐treated mice displayed the same frequency and number of OVA^+^ immune cells (Figure 6C‐D) indicating similar access of orally administered OVA to the LP. In addition, the mean fluorescence intensity (MFI) of OVA 647, reflecting the amount of OVA phagocytosed, was not significantly altered by VNS (Figure 6E). Use of CX3CR1^GFP/+^ mice reporter mice facilitated the identification of different subsets of CX3CR1 expressing macrophages and other phagocyting cells. Analysis revealed OVA was differentially distributed in some of these subpopulations between VNS‐ and sham‐stimulated mice. VNS‐treated mice displayed a significantly lower percentage and number of OVA^+^ CD11b^hi^CX3CR1^−^ cells, while displaying a higher percentage and number of OVA^+^ CD11b^hi^CX3CR1^hi^ cells. Based on the forward scatter (FSC) vs side scatter (SSC) plot, these populations are most likely eosinophils and CX3CR1^hi^ macrophages, respectively. No statistically significant changes were observed in the other OVA^+^ populations (i.e CD11b^lo^CX3CR1^−^, CD11b^int^CX3CR1^lo^ and CD11b^int^CX3CR1^int^). Additionally, no differences were observed in the mean fluorescent intensity of the OVA A647 signal, indicating that VNS does not alter the amount of OVA phagocytosed by these different populations. Taken together, these data show that VNS does not influence the overall access of antigen to the total immune cell population but instead induces an increased uptake of OVA by CX3CR1^hi^ macrophages, while reducing the uptake by eosinophils. Considering that both of these populations are capable of processing and presenting antigens, this difference in distribution might have implications on the resulting immune response and potentially contribute to the beneficial effect of VNS.

## 4 DISCUSSION

Due to its proven anti‐inflammatory properties and dense innervation of the gut, the VN has become a major topic of interest in the treatment of several gastrointestinal disorders. This anti‐inflammatory effect was initially evaluated in inflammatory models that were characterized by activation of the innate immune system. In recent years however, its potential has also been shown in diseases driven by an adaptive immune response, including Th1/Th17 colitis. In contrast, food allergy, which is characterized by a Th2 immune response and associated with gastrointestinal symptoms, has remained largely uninvestigated in this context. In this study, we therefore aimed to investigate the role of the VN in an experimental model of IgE‐mediated food allergy driven by a Th2 immune response, evaluating the impact of both a reduced and increased vagal input by means of VGX and VNS, respectively. Loss of intestinal immunological tolerance towards food antigens is typically associated with food allergy. Considering the importance of the VN in the development of oral tolerance, we hypothesized that loss of vagal input could increase the susceptibility and severity of food allergy.^15^ However, performing VGX in an experimental model of food allergy did not significantly affect the development of diarrhoea nor did it lead to significant increases in inflammatory parameters. The use of an adjuvant to break oral tolerance induces a potent activation of the immune system. We therefore speculate that the resulting inflammatory response reaches a ceiling effect that cannot be further potentiated by VGX, concluding that the basal vagal tone does not play a crucial role under these conditions.

Conversely, we hypothesized that potentiation of the anti‐inflammatory properties of the VN through VNS could decrease susceptibility and severity of food allergy. In this study, we indeed showed that application of VNS significantly improved several disease‐associated parameters, including stool scores, MC activation and numbers, serum levels of OVA‐specific IgE antibodies and gene expression levels of Th2‐associated and inflammatory genes. Several studies have previously described the α7nAChR as a critical receptor involved in the anti‐inflammatory effect of the VN.^7^, ^17^, ^18^, ^19^ Of interest, agonists of the α7nAChR, nicotine and GTS‐21, were recently shown to have anti‐inflammatory properties in a murine food allergy model.^20^ Surprisingly, we failed to support a critical role for the α7nAChR in our observed therapeutic effect of VNS. Even in α7nAChR^−/−^ mice, VNS could still significantly improve inflammatory parameters in a similar fashion as was seen before in WT Balb/C mice. These data suggest the involvement of other receptors, a finding that requires further investigation.

Immune cells, including eosinophils, MCs, ILC2s and T‐cells, express a variety of neurotransmitter receptors by which they can be modulated by the nervous system. Uncovering which immune cells are targeted by VNS in our experimental model of food allergy would allow us to unravel the underlying mechanisms by which the VN exerts its anti‐inflammatory effect. MCs in particular appeared to be consistently affected by VNS throughout our experiments. Not only were they reduced in number, as was evident in tissue sections and by flow cytometry analysis, their activation was also dampened in response to VNS, as reflected in a reduction in mMCP‐1 and IL‐4. Interestingly, MCs have been recognized as key effector cells in food allergy, indispensable for the development of symptoms like diarrhoea and systemic anaphylaxis.^21^, ^22^ They are also shown to closely interact with nerve fibres in the SI and colon, further favouring MCs as the ideal target cell for VNS.^20^, ^23^, ^24^ In addition to MCs, we also demonstrated that VNS significantly reduced infiltration of neutrophils. However, as activated MCs produce and release a range of mediators that can promote the recruitment of neutrophils to the site of inflammation, we speculate that the observed reduction in neutrophils is rather secondary to the dampening of MCs.^25^, ^26^, ^27^

Food allergy is strongly associated with altered intestinal barrier function and increased permeability. This increase in intestinal permeability is believed to be largely attributed to the activation of MCs, which will release proteases that can directly act on tight junction proteins to increase paracellular permeability.^28^, ^29^ Until recently, barrier dysfunction was therefore largely considered to be secondary to the inflammatory response. Recent insights however propose that intestinal barrier dysfunction precedes disease onset, as in inflammatory bowel disease, and may thus represent an important trigger to develop food allergy.^30^ Nonetheless, whether barrier dysfunction should be regarded as a cause or consequence of food allergy, it remains an interesting therapeutic target. The transport of immunologically intact food antigens across the intestinal epithelial barrier is indeed crucial to initiate an inflammatory response towards a specific allergen. Limiting the access of allergen by improving barrier integrity would thus represent an efficient approach by which VNS could dampen the entire immune response. Of interest, a protective effect through cholinergic modulation on barrier integrity has been previously shown in several inflammatory conditions.^31^, ^32^, ^33^, ^34^ Although application of VNS in our model of food allergy resulted in slight improvements of the TEER, this change was not statistically significant. More importantly, the improvement in TEER did not lead to differences in passage of fluorescent molecules, nor in the overall uptake of OVA by immune cells present in the SI LP. It therefore seems unlikely that VNS dampens the immune response by directly modulating barrier integrity. Oppositely, the observed dampening of MCs by VNS could prevent protease‐induced impairment of intestinal integrity and provides a more plausible explanation for the observed improvement in TEER.

The transport of OVA through the epithelial barrier was not altered in response to VNS. However, a more in‐depth investigation into the different immune cell populations that had taken up OVA revealed an interesting differential distribution of the OVA between CX3CR1^hi^ macrophages and eosinophils by VNS. Following application of VNS, more macrophages had phagocytosed OVA at cost of eosinophils. This is particularly of interest since both cell types are known to express major histocompatibility complex (MHC) II and thus able to present antigens, albeit triggering an entirely different immune response. CX3CR1^hi^ macrophages are especially known for their key role in maintaining immune homeostasis and oral tolerance by stimulating the proliferation of Treg cells.^35^ Of note, in response to cholinergic agonist, intestinal macrophages were shown to exhibit augmented phagocytosis, supporting our observations. Moreover, this effect was mediated by α4β2nAChR rather than α7nAChR.^36^ In contrast, eosinophils are typically considered as specialized effector cells during type 2‐mediated immune responses and constitute an integral cell population in the intestine, unlike in most other lymphoid and nonlymphoid tissues.^37^ Moreover, antigen‐pulsed eosinophils are able to function as antigen‐presenting cells promoting the induction and expansion of Th2 cells in several type 2 inflammatory conditions.^38^, ^39^, ^40^, ^41^, ^42^ It should be noted, though, that under healthy conditions eosinophils can actively contribute to gut immune homeostasis.^43^ In light of this, it is tempting to speculate that the increased phagocytosis of OVA by anti‐inflammatory CX3CR1^hi^ macrophages in response to VNS could be an additional mechanism by which VNS is able to dampen the immune response in our experimental model of food allergy.

In conclusion, we showed that application of VNS in an experimental model of food allergy is able to efficiently dampen the inflammatory response and improve disease outcome in an α7nAChR independent manner. Our findings further highlight the intricate relationship between the immune system and the nervous system. Further studies unravelling the exact underlying molecular mechanism and target cells are however required to provide important insights for the development of novel therapeutic strategies that will not only benefit food allergy but also other inflammatory conditions.

## CONFLICTS OF INTEREST

The authors declare that they have no conflicts of interest.

## AUTHOR CONTRIBUTIONS

GB, GM and GEB were responsible for the conception of the project and the experimental design. GB performed the experiments, analysed the data and wrote the manuscript. IA, NS, PJG‐P, MF and JAL provided assistance during experimental procedures. GM and GEB supervised the project and edited the manuscript. All authors provided critical review and final approval of the manuscript.
