# Supplementary material for: Vagus nerve stimulation dampens intestinal inflammation in a murine model of experimental food allergy
Source: Allergy. 2019 Apr 15;74(9):1748–59. doi: 10.1111/all.13790 (PMC6790670; doi:10.1111/all.13790)
Supplement: Supplementary file 1 [file ALL-74-1748-s001.pdf]

SUPPORTING INFORMATION - Vagus Nerve Stimulation dampens intestinal inflammation in a murine model of experimental Food Allergy.

Supporting Methods

Histochemistry. For histological analysis, duodenal tissue was collected and fixed in 10% formalin. 5 µm sections were stained for chloroacetate esterase (CAE) activity in MCs using a Naphthol AS-D Chloroacetate Kit (Sigma-Aldrich) according to manufacturer's instructions.

LP mononuclear cell isolation. Small intestines were cut longitudinally and epithelial cells were removed by incubating the tissue in HBSS with 5mM of EDTA and DTT and washed with HBSS. The tissue was then cut into small pieces and further digested with 2.4 mg/m collagenase A and 0.2 mg/mL DNaseI (Roche) for 30 min at 37 °C. After Percoll density gradient centrifugation, the total number of cells per intestine was determined.

Supporting tables

Table S1. Antibody panels used for flow cytometry

Antibody	Conjugate	Clone	Supplier
**Panel 1: Mast Cells**			
CD45	APC-eFluor® 780	30-F11	eBioscience
FcεRIα	PE	MAR-1	eBioscience
CD117	APC	2B8	BD Pharmingen
CD49b	PerCP-eFluor® 710	DX5	eBioscience
**Panel 2: Eosinophils and Neutrophils**			
CD45	APC-eFluor® 780	30-F11	eBioscience
Siglec-F	PE	E50-2440	BD biosciences
CD11b	PE-Cy7	M1/70	BD Pharmingen
Ly6G	PerCP-Cy5.5	1A8	BD Pharmingen
**Panel 3: ILC2s**			
CD3	Biotin	145-2C11	eBioscience
CD45R/B220	Biotin	RA3-6B2	eBioscience
CD11b	Biotin	M1/70	eBioscience
TER-119	Biotin	TER-119	eBioscience
Ly6G	Biotin	RB6-8C5	eBioscience
CD11c	Biotin	N418	eBioscience
NK1.1	Biotin	PK136	eBioscience
TCR γδ	Biotin	eBioGL3	eBioscience
CD19	Biotin	eBio1D3	eBioscience
CD5	Biotin	53-7.3	eBioscience
FcεRI	Biotin	MAR-1	eBioscience
CD45	APC-eFluor® 780	30-F11	eBioscience
CD90.2	PerCP-eFluor® 710	30-H12	eBioscience
CD25	PE	PC61.5	eBioscience
KLRG1	APC	2F1	eBioscience
**Panel 4: Tcells**			
CD45	APC-eFluor® 780	30-F11	eBioscience
CD4	APC	RM4-5	eBioscience
CD3ε	PerCP-Cy5.5	145-2C11	BD Biosciences
**Panel 5: OVA phagocyting immune cells**			
CD45	PerCP-Cy5.5	104	BD Pharmingen
CD11b	Pe-Cy7	M1/70	BD Pharmingen

Table S2. Primer sequences used for quantitative RT-PCR

Gene	Sense	Antisense
Rpl32	5'-AAGCGAAACTGGCGGAAAC-3'	5'-TAACCGATGTTGGGCATCAG-3'
Il4	5'-GGCATTTTGAACGAGGTCACA-3'	5'-GACGTTTGGCACATCCATCTC-3'
Il5	5'-CTCGTCCTCCGTCTCTCCTC-3'	5'-AAATGCTATTCCAAAACCTGTCA-3'
Il13	5'-ATGGCCTCTGTAACCGCAAG – 3'	5'-GGCTGGAGACCTGTGAAACG-3'
Il6	5'-CCATAGCTACCTGGAGTACATG-3'	5'-TGGAAATTGGGGTAGGAAGGAC-3'
Gata3	5'-CTCGGCCATTCGTACATGGAA-3'	5'-GGATACCTCTGCACCGTAGC-3'
Il25	5'-CGGAGGAGTGGCTGAAGTGGAG-3'	5'-ATGGGTACCTTCCTCGCCATG-3'
Tslp	5'-TCGAGGACTGTGAGAGCAAGCCAG-3'	5'-CTGGAGATTGCATGAAGGAATACCA-3'
Tnfa	5'-TCTTCTCATTCCTGCTTGTGG-3'	5'- CACTTGGTGGTTTGCTACGA-3'
